# Acute aortic dissection type A discloses Corpus alienum

**DOI:** 10.1186/1749-8090-4-1

**Published:** 2009-01-02

**Authors:** Aron Frederik Popov, Mersa Mohammed Baryalei, Jan Dieter Schmitto, Jose Hinz, Christoph Hermann Wiese, Björn Raab, Philipp Kolat, Friedrich Albert Schoendube, Ralf Seipelt

**Affiliations:** 1 Department of Thoracic and Cardiovascular Surgery, University of Göttingen, Germany; 2 Department of Anaesthesiology, Emergency and Intensive Care Medicine, University of Göttingen, Germany; 3 Department of Radiology, University of Göttingen, Germany

## Abstract

We report an unusual case of an aortic type A dissection with a corpus alienum which compresses the right ventricle. The patient successfully underwent an aortic root replacement in deep hypothermia with re-implantation of the coronary arteries using a modified Bentall procedure and the resection of the corpus alienum. Intraoperative finding reveals 3 greatly adhered gauze compresses, which were most likely forgotten in the operation 34 years ago.

## Case presentation

**Figure 1:**
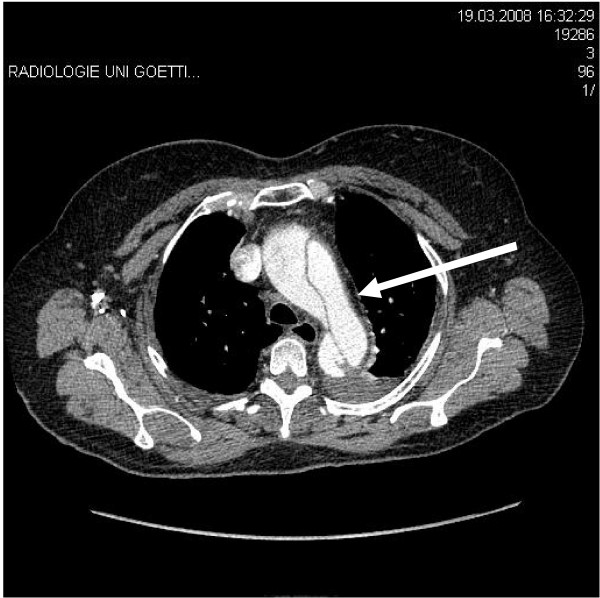
**CT-scan showing type A dissection with a dissection flap**.

**Figure 2:**
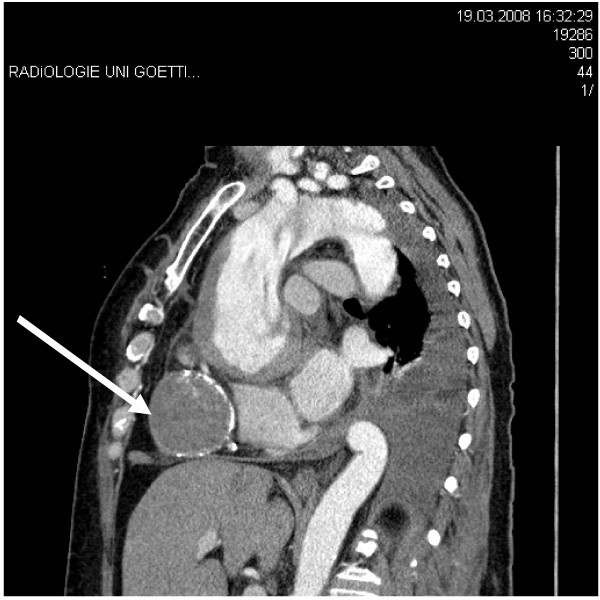
**CT scan showing an adherent structure which compressing the right ventricle**.

**Figure 3:**
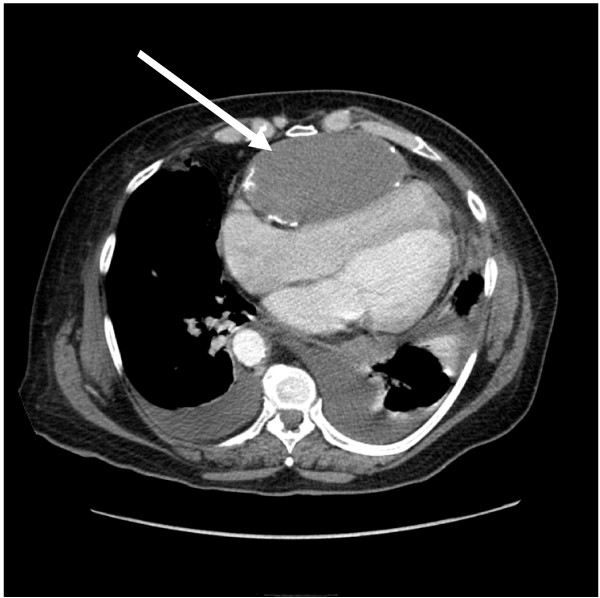
**CT scan showing an adherent structure which compressing the right ventricle**.

A 64-year-old patient was referred to us from another hospital with symptoms of angina pectoris and suspicion of constrictive pericarditis. An acute myocardial infarction had already been ruled out. Further diagnostic measures were carried out in our hospital. Due to increasing pain and hemodynamic instability, a thorax computed tomography (CT) with contrast medium was performed. This revealed a type A dissection with suspicion of a bloody pericardial effusion (Figures 1, 2 and 3). The patient had been healthy up to this day, only in 1974 at the age of 30, an ASD closure had been carried out via lateral thoracotomy for an atrial septum defect. The operation was carried out at another institution and postoperative course was uneventful.

**Figure 4:**
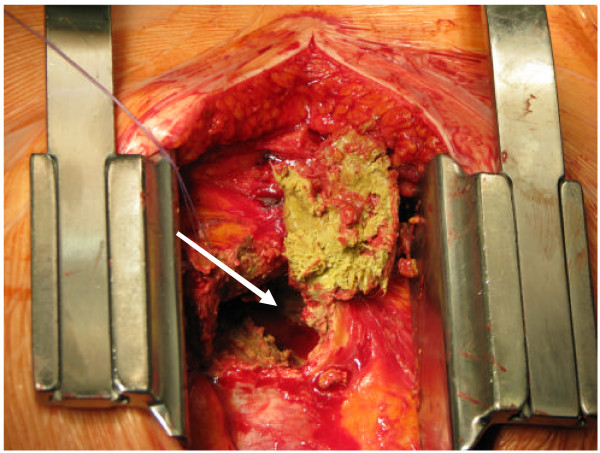
**Intraoperative view of the opened pericardium with purulent structure**.

**Figure 5:**
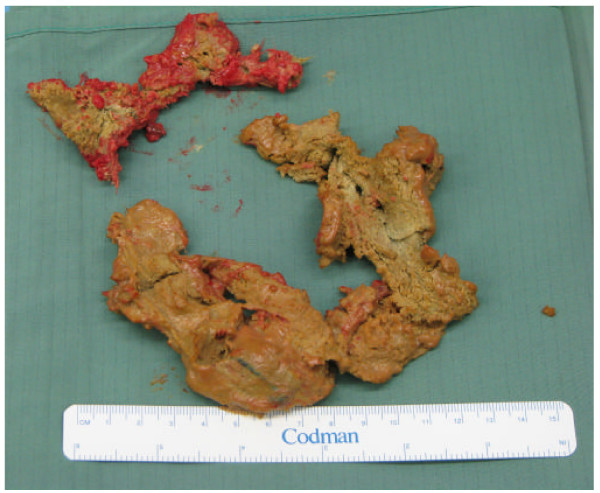
**Three gauze compresses which were secured**.

The emergency operation was initiated immediately after the diagnosis was made. The patient was connected to the heart-lung machine via the groin. A sternotomy was performed, revealing an adherent purulent structure (Figure 4), approximately 4–5 cm dimension that was clearly compressing the right ventricle without pericardial effusion, located in close relation to the ascending aorta. After cautious dissection, 3 greatly adhered gauze compresses (Figure 5) were carefully removed from the pericardium. These were most likely forgotten in the operation 34 years ago. Due to this finding, the emergency surgery was aborted due to the infectious process.

The patient was transferred to the intensive care ward and discharged on the 10th postoperative day without treatment of the type A dissection. Postoperative microbiological tests showed no indication of bacterial colonization. During the hospital stay we started an antihypertensive therapy. However, after the first operation at our institute the patient wanted time to think it over concerning the forthcoming high-risk operation. Although we insistently informed the patient about the dangerous situation, she contacted us not until five weeks with the decision that we can perform the operation.

The patient was rescheduled six weeks later after the first operation at our institute to repeat the CT-scan. This showed a progression of the type A dissection with renewed hemorrhagic pleural effusion followed by promptly surgical treatment.

**Figure 6:**
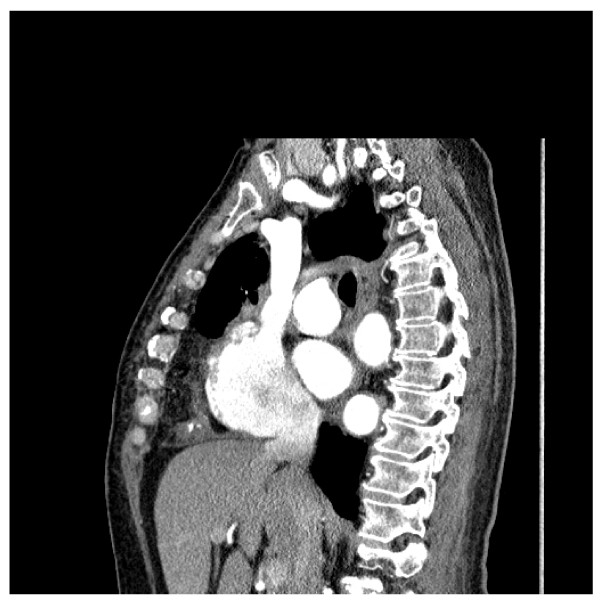
**Follow-up CT-scan after one month with a decompressed right ventricle**.

**Figure 7:**
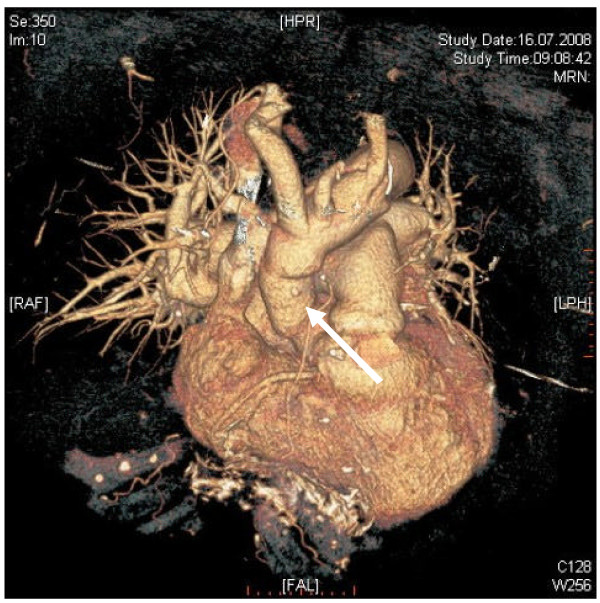
**Three-dimensional volume rendered image showing the postoperative result after one month**.

Connection to the heart-lung machine was again through the groin and the thorax was reopened again. An aortic root replacement in deep hypothermia with re-implantation of the coronary arteries using a modified Bentall procedure was carried out. The aortic valve was replaced by a Medtronic Stentless Freestyle bioprosthesis. The supra-aortic vessels were re-implanted and the ascendeing aorta and aortic arch were replaced with a Hemashield-prosthesis. The postoperative course was unremarkable and the patient was discharged after 14 days. A follow-up CT-scan was carried out one month later that documented a continued good postoperative result with a decompressed right ventricle (Figures 6 and 7). The patient is free of pain and able to function well in daily life.

## Discussion

Acute type A aortic dissection is a catastrophic disease that requires immediate surgical intervention. The main goal of surgery in acute type A aortic dissection is to prevent death from intrapericardial hemorrhage by resecting and replacing the diseased aorta with a graft [1]. Aggressive surgical approach involving extensive resection of dissected aorta for patients with aortic dissection became more popular over the recent years [2]. However, in this case the surgical treatment was deferred, because the adhered gauze compresses was to be suspected infectious process. Moreover, it is well known that infections involving ascending aortic grafts are extremely difficult to eradicate and are frequently lethal [3]. Treatment of this complication remains a challenge for surgeons, and chances of a successful outcome are considered low. Mortality rates range from 25% to 75%, and morbidity in surviving patients is high [4]. Several groups currently favour replacing infected ascending aortic prostheses with cryopreserved aortic homografts [4-7]. Retrospective data have suggested that, compared with using synthetic grafts, using cryopreserved homografts for treating vascular infections is associated with improved outcomes, including better elimination of infection, fewer postoperative complications, and longer disease-related survival [8]. However, one commonly cited disadvantage of using homografts is their predisposition to progressive deterioration and ultimate need for re-replacement [9,10]. Unfortunately, in many cases, a single homograft will not reach the distal ascending aorta or transverse arch [7]. Extensive aortic replacement can be accomplished by using total arch homografts, but these are rarely available [11]. Another treatment strategy to prevent recurrent infection is using a pedicled omental or muscle flap. Omentum is particularly popular because, in patients who have not had previous abdominal surgery, omentum can be easily accessed by extending the sternotomy incision into the abdomen for a short distance [12-16]. The blood supply to the omentum is preserved by basing the pedicle on the right gastroepiploic artery. In addition to filling dead space, the vascularised omental pedicle improves oxygen supply to the region, enhances immunologic response, increases antibiotic delivery, and absorbs wound secretions that can serve as substrates for bacterial growth [13,16].

There are, however, many established options for managing the infected graft, but the literature on surgical treatment of ascending aortic graft infection fails to provide even the lowest level of evidence on which to base a concrete recommendation. Otherwise, the patient was not septic on admission. However, at that point of time the intraoperative situation was not clear concerning the potential infection, we decided in this special case to abort the emergency operation.

## Conclusion

Delayed surgical treatment could be an acceptable alternative to prompt intervention in aortic dissection, provided a stable clinical condition can be achieved with conservative management consisting of rest, careful monitoring and periodic reassessment, if other conditions make the immediate operation very dangerous.

## Consent

Written informed consent was obtained from the patient for publication of this case report and any accompanying images. A copy of the written consent is available for review by the Editor-in-Chief of this journal.

## Competing interests

The authors declare that they have no competing interests.

## Authors' contributions

AP, MB, RS, JS and PK are members of surgical teams. CW and JH were the anaesthetists involved in theatre and in intensive care unit. BR was the radiologist. FS co-wrote the manuscript and added important comments to the paper. All authors have read and approved the final manuscript.
